# Improving Cross-Day EEG-Based Emotion Classification Using Robust Principal Component Analysis

**DOI:** 10.3389/fncom.2017.00064

**Published:** 2017-07-19

**Authors:** Yuan-Pin Lin, Ping-Keng Jao, Yi-Hsuan Yang

**Affiliations:** ^1^Institute of Medical Science and Technology, National Sun Yat-sen University Kaohsiung, Taiwan; ^2^Institute for Neural Computation, University of California, San Diego La Jolla, CA, United States; ^3^Center for Neuroprosthetics, School of Engineering, École Polytechnique Fédérale de Lausanne Geneva, Switzerland; ^4^Research Center for IT Innovation, Academia Sinica Taipei, Taiwan

**Keywords:** inter-day EEG variability, emotion classification, affective brain-computer interface, EEG oscillation, robust principal component analysis

## Abstract

Constructing a robust emotion-aware analytical framework using non-invasively recorded electroencephalogram (EEG) signals has gained intensive attentions nowadays. However, as deploying a laboratory-oriented proof-of-concept study toward real-world applications, researchers are now facing an ecological challenge that the EEG patterns recorded in real life substantially change across days (i.e., day-to-day variability), arguably making the pre-defined predictive model vulnerable to the given EEG signals of a separate day. The present work addressed how to mitigate the inter-day EEG variability of emotional responses with an attempt to facilitate cross-day emotion classification, which was less concerned in the literature. This study proposed a robust principal component analysis (RPCA)-based signal filtering strategy and validated its neurophysiological validity and machine-learning practicability on a binary emotion classification task (happiness vs. sadness) using a five-day EEG dataset of 12 subjects when participated in a music-listening task. The empirical results showed that the RPCA-decomposed sparse signals (RPCA-S) enabled filtering off the background EEG activity that contributed more to the inter-day variability, and predominately captured the EEG oscillations of emotional responses that behaved relatively consistent along days. Through applying a realistic add-day-in classification validation scheme, the RPCA-S progressively exploited more informative features (from 12.67 ± 5.99 to 20.83 ± 7.18) and improved the cross-day binary emotion-classification accuracy (from 58.31 ± 12.33% to 64.03 ± 8.40%) as trained the EEG signals from one to four recording days and tested against one unseen subsequent day. The original EEG features (prior to RPCA processing) neither achieved the cross-day classification (the accuracy was around chance level) nor replicated the encouraging improvement due to the inter-day EEG variability. This result demonstrated the effectiveness of the proposed method and may shed some light on developing a realistic emotion-classification analytical framework alleviating day-to-day variability.

## Introduction

Implicit emotional reactions behave as a non-verbal psychophysiological communication channel alternative to explicit manners of body gestures, written text, and speech, enriching the interaction in people. Through characterizing such emotional information by leveraging multidisciplinary knowledge and the ever-growing affective computing technology, a conventional human-computer interaction (HCI) scenario can then be augmented with an emotion-aware ability, which facilitates a realistic humanoid closed-loop feedback. Emotion recognition has attracted intensive attention nowadays. Two facets may drive its intensive interest. On one hand, it enables a wide spectrum of intriguing emotion-oriented applications such as, machine intelligence (Chen et al., 2017), receptionist robots (Pinheiro et al., 2017), content recommendation devices (Lee and Shin, 2013), tutoring systems (Muñoz et al., 2010), and music therapy (Ian et al., 2016). On the other hand, recent explosive innovations in wearable sensing technology considerably bring laboratory-demonstrated emotion-aware research closer to our daily life, necessitating a robust and accurate emotion-aware analytical framework.

Existing affective computing research has demonstrated the capacity of assessing implicit emotions by internal changes in physiological signals that are originated from autonomic and central nervous systems (Picard et al., 2001; Kim and Andre, 2008; Chanel et al., 2009; Lin et al., 2010). Among them, electroencephalogram (EEG) is a non-invasive-recording of the electrical activity of the brain. The EEG signals presumably encompass the fundamental yet critical information underlying emotion dynamics, since the limbic system located in the brain plays a key role in emotion regulation (Hariri et al., 2000). There has been promising interdisciplinary analytical frameworks proposed to leverage advanced signal processing, machine learning, and data mining techniques with the attempt to exploit the EEG correlates of emotional responses as well as to later develop an emotion-aware model for emotion recognition (Chanel et al., 2009; Frantzidis et al., 2010; Lin et al., 2010; Petrantonakis and Hadjileontiadis, 2010; Koelstra et al., 2012; Soleymani et al., 2012; Jenke et al., 2014; Gupta et al., 2016; Hu et al., 2017). This area has become an emerging track in the affective brain-computer interface (ABCI), namely EEG-based emotion recognition (Mühl et al., 2014). The successful demonstrations would not only demonstrate the feasibility of emotional computing from EEG signals, but also pose new directions for practical ABCI applications in real life.

A practical issue for exploiting the EEG correlates of implicit emotional responses is about how many EEG samples are needed from an individual to reliably model the emotional responses. The issue has also been recognized as a plausible factor affecting the classification accuracy while training a machine-learning classifier upon the given data. The previous study results may support the argument in part. The works that involved a short-duration (around 1–15 s per trial) emotion elicitation scenario, e.g., image viewing and emotion imagery (Chanel et al., 2009; Frantzidis et al., 2010; Petrantonakis and Hadjileontiadis, 2010), typically led to better results than those involved a long-duration manner (around 30–120 s per trial), e.g., music listening and video watching (Koelstra et al., 2012; Soleymani et al., 2012; Gupta et al., 2016). In practice, an emotion experiment with EEG recordings faces a trade-off between acquiring more data trials and preventing the human subjects from being bored and drowsy to elicitation materials. In most cases, fewer than a few dozen of trials per targeted emotional class can be collected in a 2~3-h experiment session for an individual, including the time for instruction briefing and EEG headset capping. The collected EEG trials are thus rare and likely pose a challenge for translating the EEG spatio-spectral oscillations into implicit emotional responses and for utilizing the EEG-emotion relationship to train a realistic subject-specific emotion-classification model.

A straightforward remedy for the aforementioned challenge in a single-day session is to perform a multiple-session EEG recording on separate days. Nevertheless, this raises another issue concerning the substantial inter-day variability in the EEG signals, which has been empirically demonstrated in studies (Christensen et al., 2012; Lin et al., 2015; Das et al., 2016; Yin and Zhang, 2017). That is, EEG features recorded on different days were found distinctively distributed. The data clusters of the same classes across days happened to behave more diversely than the clusters of different classes within a single day (Lin et al., 2015). This finding was in line with the outcomes using peripheral bio-signals (Picard et al., 2001). The class clusters were even dramatically changed by reversal among different days (Christensen et al., 2012). As such, the day-to-day variability inevitably hindered a machine-learning classifier from leveraging an effective set of between-class decision boundaries that can work consistently to the data recorded across days. It might happen that naively aggregating the EEG samples from all of the available recording days degrades rather than upgrades the classification accuracy (Christensen et al., 2012; Lin et al., 2015). Few attempts have been made to alleviate the inter-day variability by either using different normalization schemes or seeking a set of relatively day-robust features in other EEG topics, e.g., cognitive load, mental workload, and biometrics (Christensen et al., 2012; Das et al., 2016; Yin and Zhang, 2017). Till now, most of previous analytical works (Chanel et al., 2009; Frantzidis et al., 2010; Lin et al., 2010; Petrantonakis and Hadjileontiadis, 2010; Koelstra et al., 2012; Soleymani et al., 2012; Jenke et al., 2014; Gupta et al., 2016; Hu et al., 2017) endeavored to optimizing a predicative emotion-aware model based on a non-ecological single-day dataset only. However, for an ecological ABCI scenario, the EEG signals may vary over time, leading to the alternation of the emotion-related EEG oscillations, and thereby making a model trained by the EEG signals of a separate day(s) vulnerable. Relatively fewer efforts have been contributed to thoroughly explore and tackle the impact of the inter-day EEG variability associated with emotional responses, which is believed to be one of critical factors hindering the success of real-life applications.

To address the issue mentioned above, this work proposed a signal-filtering strategy based on a core methodology called robust principal component analysis (RPCA) and incorporated it into a machine-learning framework. Its capability was demonstrated in terms of cross-day emotion-classification results and corresponding neurophysiological meanings through a 5-day EEG dataset of 12 subjects. RPCA behaves as a matrix factorization method and enables parsing the input data matrix into a low-rank matrix and a sparse matrix. The low-rank matrix represents relatively regular activity or patterns in the original input matrix, whereas the sparse matrix accounts for deviant events. RPCA has been applied to improve the tracking of sparse moving targets of interest in video surveillance (Guyon et al., 2012; Bouwmans and Zahzah, 2014) and recently applied to affective computing to better capture the EEG correlates of neurocognitive lapses (Wei et al., 2016) as well as emotional responses (Jao et al., 2015). It is worth noting that this work was an extension from our proof-of-concept study (Jao et al., 2015) with considerable improvement in two aspects. First, this work provided neurophysiological evidence to exclusively elucidate the meanings underlying RPCA decomposition in emotion data. Second, a simulated online BCI validation procedure, i.e., training the data from available days and testing on the data from an unseen day, was employed to assess the cross-day classification performance regarding the accuracy and the number of informative features exploited. The successful demonstration can shed some light on developing a realistic emotion-classification analytical framework accounting for the EEG discrepancy in separate days.

## Materials and methods

### EEG dataset

**Figure 1:**
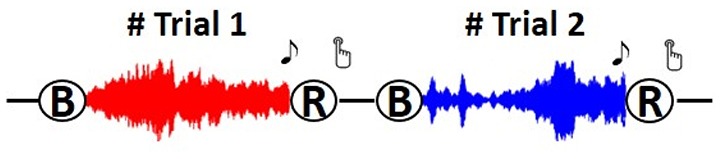
The experiment protocol of a two-trial block. Each trial begins with a 15-s baseline (B), followed by a ~37-s music excerpt, and ended with an emotion-tagging task (R). A beep sound was delivered to alert subjects for the rating task. The protocol was self-paced and proceeded to next trial after the subject pressed the button.

This work assessed the practicability of RPCA framework and its underlying neurophysiological meanings in alleviating the inter-day EEG variability on a 5-day dataset of 12 subjects when they performed a music-listening task (Lin et al., 2015). The details regarding the music excerpts and experiment setup can be found in Lin et al. (2015). Briefly, a 14-channel Emotiv EEG headset, with a default bandwidth of 0.16–43 Hz and a sampling rate of 128 Hz, was employed to measure the EEG signals. The subjects participated in the same music listening experiment on 5 different days within one and half weeks (with an average interval of 7 ± 1.13 days). On each day, they underwent a three-session protocol composed of the same 24~37-s music excerpts to induce two target emotions, happiness and sadness, in which 12 excerpts for each category were selected with a consensus label (Eerola and Vuoskoski, 2011). Each session had four blocks; each of them contained both happy and sad trials in random order. Figure 1 illustrates the procedures of a two-trial block. Each trial began with a 15-s eye-closed rest period, followed by a music excerpt. A beep sound alerted the subjects to proceed to an emotion-assessment task (assigned either one of target emotions or neutral based on whey they experienced). The experiment was self-paced and allowed the subject to press a button proceeding to the next trial, enabling a moderate rest if necessary. Such an experiment protocol collected 24 pairs of ~37-s EEG trials (plus a 15-s eye-closed baseline) and emotion labels from an individual in each of the 5 recording days. Note that the trials reported as neutral responses were excluded from further analysis.

### EEG feature extraction

The raw EEG signals were first submitted to a 1-Hz high-pass finite impulse response filter to remove possible DC drifts. The short time Fourier transform was then adopted to estimate the spectral power of the filtered EEG signals using a 1-s Hamming window with a 50% overlap, yielding a number of the samples depended on the time lapse of the given trial (~37-s). The averaged band power over the stereotypical frequency bands of delta (1–3 Hz), theta (4–7 Hz), alpha (8–13 Hz), beta (14–30 Hz), and gamma (31–43 Hz) was calculated prior to feature extraction. Note that Emotiv headset's specification limited the gamma frequency band up to 43 Hz.

**Figure 2:**
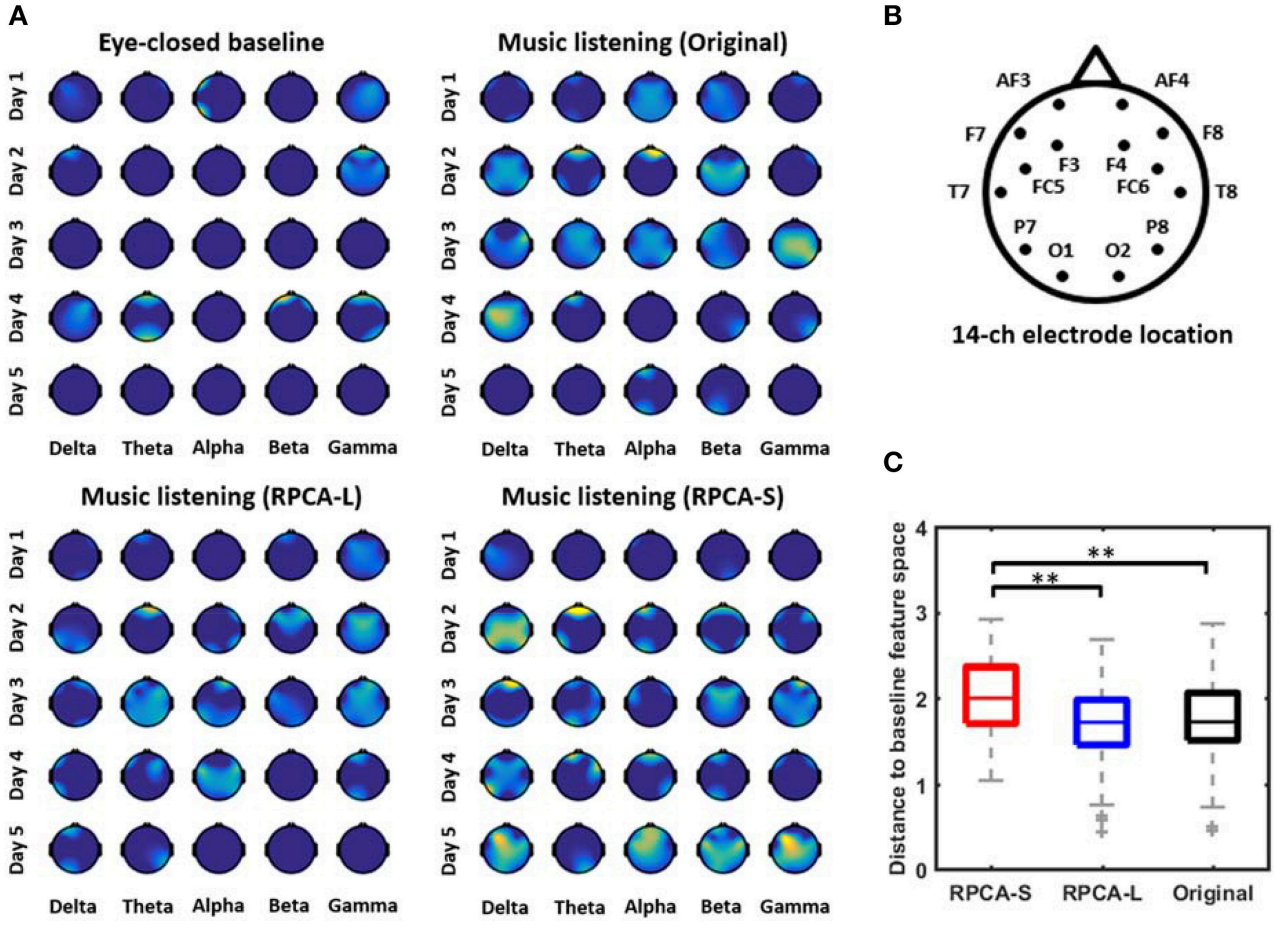
The single-day emotion-related topographic feature maps with and without RPCA preprocessing. **(A)** illustrates the informative maps of a representative subject derived separately by four analytical manners, including EEG signals in eye-closed resting baseline, EEG signals in music listening (Original), RPCA-processed sparse matrix (RPCA-S) in music listening, and RPCA-processed low-rank matrix (RPCA-L) in music listening. The importance of the features was normalized from 0 to 1 and color-coded from dark blue to brighter yellow, accordingly. The brighter yellow reflected the feature more informative with respect to dark blue (no correlation). **(B)** Refers to the electrode montage. **(C)** The Euclidean distance for topographic outcomes between RPCA-S/RPCA-L/Original vs. eye-closed resting baseline summarized from 12 subjects. The longer distance indicated most informative EEG dynamics captured by the analytical manner with a reasonable assumption that the resting baseline was associated with a minimal correlation with emotional responses. ^**^ Refers to a statistical significance with *p* < 0.01 using a two-sided Wilcoxon signed rank test.

Given the five time series of spectral bands, this study adopted a feature-extraction method called MESH (Lin et al., 2014) to correlate EEG spectral oscillations with emotional responses. The MESH method not only includes the spectral oscillation over individual electrodes but also assesses bi-directional power asymmetry over left-right symmetric electrodes (i.e., laterality) and fronto-posterior electrodes (i.e., caudality). As such, the 12 channels (excluding T7 and T8 from the 14-ch Emotiv montage) corresponded to six left-right electrode pairs (i.e., AF3–AF4, F7–F8, F3–F4, FC5–FC6, P7–P8, and O1–O2, the montage refers to Figure 2B) and four fronto-posterior pairs (i.e., AF3–O1, F7–P7, AF4–O2, and F8–P8), resulting in a feature dimension of 110 (22 electrode attributes × five frequency bands). Each feature time series was normalized to the range of 0 and 1 using the min-max normalization scheme.

### Robust principal component analysis (RPCA)

Unlike classical principal component analysis (PCA) that transforms signals into a set of mutually orthogonal variables for dimensionality reduction, RPCA is a matrix factorization method that decomposes an input matrix *X* ϵ R^*m*^^×*n*^ (*m*: number of features, *n*: number of samples) into two superimposed matrices, a low-rank matrix *L* and a sparse matrix *S* (Candes et al., 2011). The *L* accounts for the relatively regular profiles of input signals, whereas the *S* models its deviant events. The RPCA can be mathematically described as the convex optimization problem (Candes et al., 2011) presented below:

(1) $$\min{\;}_{L,S}∥L\;{∥}_{*}+\lambda ∥S{∥}_{1}\;s.t.\;\;X=L+S\;,$$

where ∥*L* ∥_*_ denotes the nuclear norm of the matrix *L*, i.e., the sum of the singular value of *L*, ∥*S*∥_1_ = ∑_*ij*_|*S*_*ij*_| represents *l* -1 norm of *S*, and λ is a tuning parameter balancing the weights of the two terms; we set λ to $1/\sqrt{\max\left(m,n\right)}$ according to Candes et al. (2011). RPCA has been successfully applied to many signal processing and computer vision problems, such as, video processing (Bouwmans and Zahzah, 2014), face recognition (Chen et al., 2012), and music information retrieval (Yang, 2012) as well as a recent demonstration on affective computing using EEG signals (Wei et al., 2016). For example, in the case of video surveillance, RPCA decomposed the still background scene in *L* and encompassed the sparse moving objects in *S*. It thus facilitated the detection of moving objects of interest by eliminating the interference from the background. It is worth noting that the robust PCA algorithm proposed by Torre and Black (2003) and the RPCA algorithm (Candes et al., 2011) adopted in this work, though share similar names, are radically different in their mathematical meanings. The former is for data dimension reduction, whereas the latter is for matrix decomposition that enables to parse inputs signals into low-rank and sparse matrices.

In a music-listening study, it is reasonable to assume that EEG oscillations associated with emotional responses are considered as deviant and sparse activity concurrent with intrinsic background EEG activity. Such background EEG activity tends to be relatively regular within days yet more and less diverse across days. The non-stationary background activity may thus submerge the sparse emotion-related EEG oscillations of interest and inevitably hinder the robustness of a cross-day emotion classification framework. With this in mind, this study hypothesized that the more the emotion-irrelevant EEG perturbations can be alleviated in each day, the more the elicited emotion-related EEG oscillations can be revealed. To test the posed hypothesis, this study adopted RPCA to parse the MESH matrix (i.e., 110 features × *n* samples, where *n* depends on the number of samples given a trial) into low-rank *L* and sparse *S* matrices. The resultant *L* presumably described relatively regular background EEG activity, while the resultant *S* oppositely captured sparse emotion-related dynamics.

### EEG feature selection and classification

After applying RPCA framework to the spectral time series, the processed samples were averaged within each trial for feature selection and classification. A straightforward method of F-score feature selection was adopted to elaborate the MESH feature space (either with or without RPCA pre-processing) to exploit an optimal subset of informative features prior to training the classifier. The F-score value refers to the ratio of between-class vs. within-class variance formed by the data distribution of a feature. It has been shown that the features with high *F*-score values can better discriminate class distributions (Lin et al., 2010; Jenke et al., 2014). Most importantly, as the calculated *F*-score value was compared to the statistical *F*-distribution, the corresponding statistical *p*-value of each feature (*p* < 0.05) can be derived to elucidate the neurophysiological meanings of RPCA-decomposed low-rank and sparse matrices. In this proof-of-concept study, a simple Gaussian Naïve Bayes (GNB) classifier was employed to model the EEG data distributions along two emotion categories (i.e., happiness vs. sadness). The cross-day classification accuracy referred to how many trials were correctly classified.

### Validation of RPCA framework

This study attempted not only to test the effectiveness of the RPCA framework in alleviating the day-to-day variability in emotion-related EEG dynamics but also endeavored to unveil its underlying neurophysiological evidence. Three cross-day analytical scenarios were conceived and performed accordingly, including emotion classification, emotion-class distribution, and emotion-related spatio-spectral features.

First, a realistic add-day-in (ADI) validation framework was adopted to assess cross-day emotion classification accuracy. The ADI scheme iteratively included the data from one more recording day to train a classifier and test its performance against the data from one unseen recording day. That is, the information of EEG signals to be tested were entirely disjointed from the data used for training the model, which complied with a real-life BCI validation framework. Given a five-day EEG dataset per subject in this study, the cross-day classification accuracy can be obtained for four training day scenarios, including (1) Day 1 vs. Day 2, (2) Days 1–2 vs. Day 3, (3) Days 1–3 vs. Day 4, and (4) Days 1–4 vs. Day 5. The procedures of each ADI framework are detailed as follows.

1. Train and optimize a GNB classifier
  This step first concatenated data from *D* available training day(s) (*D* = 1–4). The concatenated data were then submitted to *F*-score feature selection to rank MESH features. To prevent the plausible bias caused by class imbalance, the GNB model was trained and optimized given 100 repetitive outcomes with random samples equally selected (according to the minimal class) from binary classes. Each randomization performed a five-fold cross-validation and an add-feature-in scheme, i.e., iteratively adding one more feature with high F-score at a time. The optimal MESH feature subspace leading to a maximal training accuracy could be selected.
2. Test the GNB classifier
  The data from an unseen recording day were treated as test samples, and its initial MESH feature space was trimmed to fit the subspace optimized in the training phase. The trained GNB model was then tested on the trimmed data.

The ADI validation framework was applied to the EEG signals leveraged without and with RPCA processing for comparison.

Second, this study additionally visualized the emotion class distributions across days following the ADI manner. In this way, the variability in EEG signals between classes across days can be explored. We adopted the linear discriminative analysis (LDA) to reduce the original feature dimensionality (110) to a 2-D discriminative yet comprehensible feature space composed of the first two LDA components. Note that the LDA was simply involved in data visualization rather than in classification task. Furthermore, this study superimposed a decision boundary over the class clusters of the training data artificially. The boundary laid perpendicular to the vector of the means of the clusters and intercepted at their center. The multiple-day class distributions plus the conceptualized decision boundaries intended to demonstrate two facts: how the inter-day variability shaped the distributions of training and testing data, and to which extent this variability behaved in EEG signals with and without RPCA preprocessing.

Last, this study mapped the emotion-related EEG features that corresponded to high *F*-score values with statistical significance (*p* < 0.05) onto topography. The topographic mapping was done by using EEGLAB toolbox (Delorme and Makeig, 2004). Through comparing the topographic outcomes between a pre-stimulus baseline, i.e., eye-closed resting state, and a music-listening period, we could somehow elucidate the neurophysiological meanings underlying the RPCA-decomposed low-rank and sparse matrices. The low-rank matrix supposedly contained mostly background EEG dynamics, so that it barely had spectral characteristics about emotional responses. Thus, the maps of low-rank matrix exploited in music listening were presumably similar to those from the resting state. Furthermore, the ADI classification was also replicated on the eye-closed baseline. The baseline-music listening comparison can directly assess the validity of RPCA processing and F-score feature selection for cross-day emotion classification with more neurophysiological sense (i.e., EEG signals without and with music elicitation). Note that for such analysis each of the pre-stimulus baseline trials was artificially assigned with an emotion label equal to the one rated right after the subsequent music-listening trial. In addition, for group analysis, this study vectorized each topographic outcome and objectively quantified their similarity using the Euclidean distance measurement. A longer distance referred to two distinct feature maps being compared.

## Results

### Single-day topographic feature maps underlying RPCA matrices

Figure 2 presents the single-day emotion-relevant EEG spatio-spectral features explored in music-listening vs. eye-closed resting scenarios. The comparative outcomes were obtained for four data matrices separately, including EEG signals in eye-closed resting baseline, EEG signals in music listening (Original), RPCA-processed sparse matrix (RPCA-S) in music listening, and RPCA-processed low-rank matrix (RPCA-L) in music listening. Figure 2A color-coded the importance (i.e., the *F*-score values) of the band-power features onto topography (c.f. the electrode montage in Figure 2B) from a representative subject. All topographic values of four analytical outcomes were normalized concurrently to the range of 0 and 1 within each day so that the extent of each feature importance of all topographic outcomes could be compared across days. The brighter yellow indicates more informative features, compared to dark blue (no correlation). As can be seen in the topographic feature maps, the RPCA-S generally exhibited more emotion-related spectral features in each band and on each day, compared to its original input, i.e., Original. In contrast, the RPCA-L simply led to minor yet fewer informative features (with lighter yellow) on certain days. The benchmark scenario of eye-closed resting barely accompanied features. Most of the resting topographies were annotated with dark blue, similar to the outcomes of RPCA-L. Note that both the RPCA-L and RPCA-S of the resting baseline got analogous outcomes irrelevant to emotional responses (but not shown here).

Figure 2C further quantified to which extent the emotion-related topographic maps with/without RPCA (i.e., RPCA-S, RPCA-L, and Original in music listening) were deviant from those of the benchmark (i.e., the eye-closed baseline) from the entire group of 12 subjects. To this end, the Euclidean distance measurement was adopted to calculate the distance between the-vectorized topographic maps within each day. A longer distance value indicated the analytical matrix of interest being most informative under a reasonable assumption that the eye-closed resting state accounted for the minimal information regarding emotional responses. As can be seen, RPCA-S differed most from the eye-closed baseline than both the Original and RPCA-L did (*p* < 0.01). Due to the shortest distance, the RPCA-L's feature maps were the most similar to the baseline, followed by the Original.

### Cross-day emotion class distributions with and without RPCA processing

**Figure 3:**
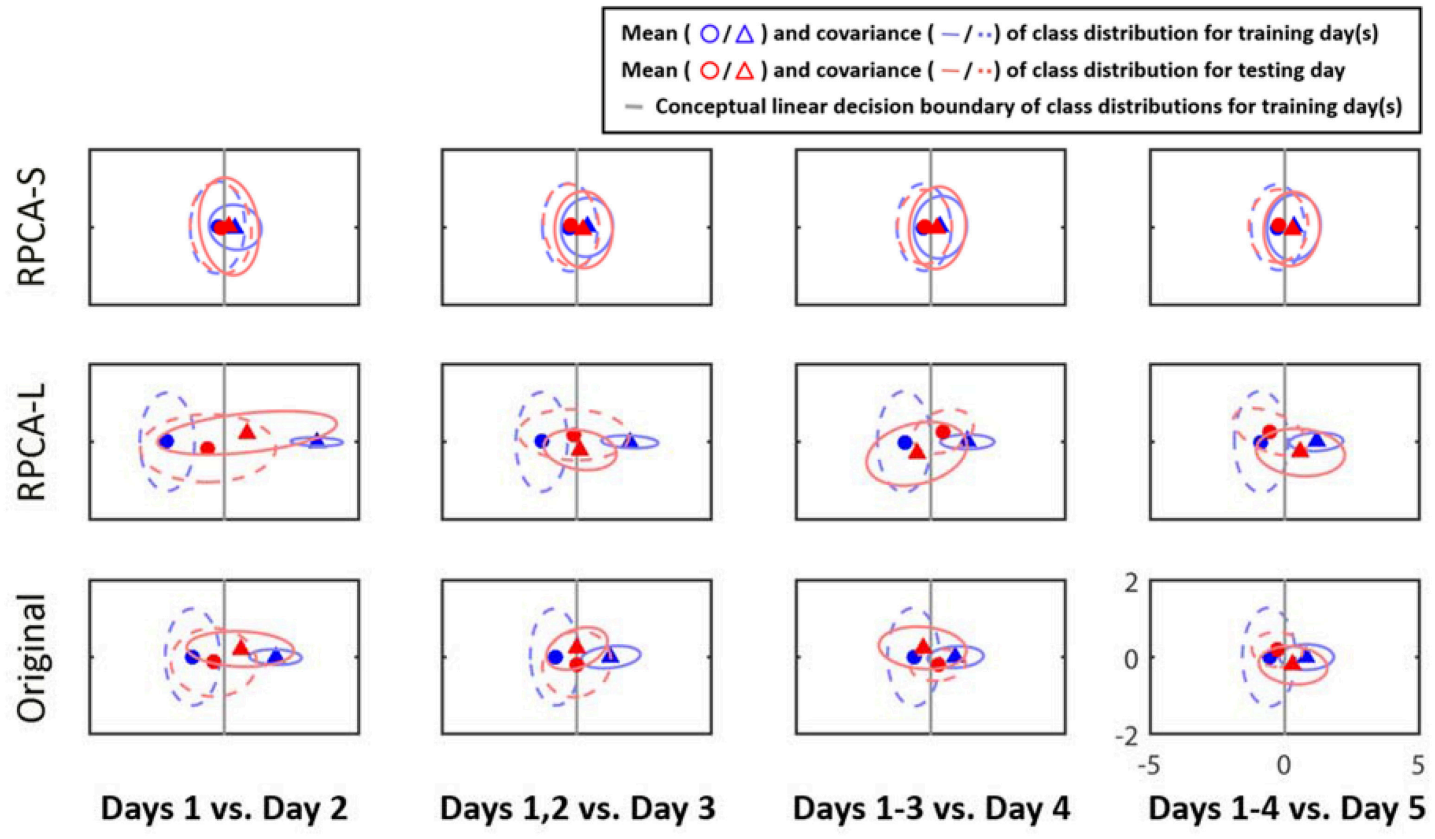
The 2D projection of the cross-day class distributions of the EEG signals by LDA for a representative subject in ADI manner. The rows of subplots indicate the outcomes leveraged with or without RPCA processing (RPCA-S, sparse matrix; RPCA-L, low-rank matrix; Original, original signals). Triangles and circles are the centers of the happiness and sadness clusters, respectively, whereas the dotted and solid ellipses reflect their covariance values. The annotations in red and in blue referred to training and test days, respectively. The gray lines conceptualize the decision boundaries of the training data distributions.

Figure 3 illustrates the class distributions of the EEG signals projected to a 2D LDA feature space from a representative subject. The row of subplots from the bottom to the top represents the distributions of the original EEG signals (Original), its RPCA's low-rank matrix (RPCA-L), and its RPCA's sparse matrix (RPCA-S). The subplots along columns show the outcome with the ADI manner. As can be seen, the inter-day variability in Original did negatively shape the class distributions of the training data based on the relationship between the class centroids and distributions as the EEG signals were taken into account from more recording days. In the case of Day 1 vs. Day 2 in Original, the decision boundary (gray line) for Day 1 seemed to work for Day 2 because the two class centroids of the two days lined aside moderately. Nevertheless, the separable class centroids became misleading as considering the training data from one more recording day, i.e., Days 1–2 vs. Day 3, and even confusing by adding more days for the condition, i.e., Days 1–3 vs. Day 4, as referenced to their decision boundaries. While involving four recording days for training (Days 1–4 vs. Day 5), there was a smaller between-class margin than that of the initial outcome (Day 1 vs. Day 2). Next, after leveraging the EEG signals with RPCA processing, the inter-day variability tended to be mitigated to a certain extent. In the RPCA-S, the interplay of the class centroids of the training and testing days remained relatively stable to all ADI conditions, i.e., invulnerable to the number of recording days involved. Importantly, the decision boundary got improved marginally yet progressively when considering the data from more days as training dataset. On the contrary, the RPCA-L resembled the Original, but exhibited larger covariance in class distributions. The above results evidently demonstrated the negative impact of the potential inter-day variability to a predictive emotion model, but the RPCA framework was capable of alleviating it to some extent.

### Cross-day emotion classification with and without RPCA processing

**Figure 4:**
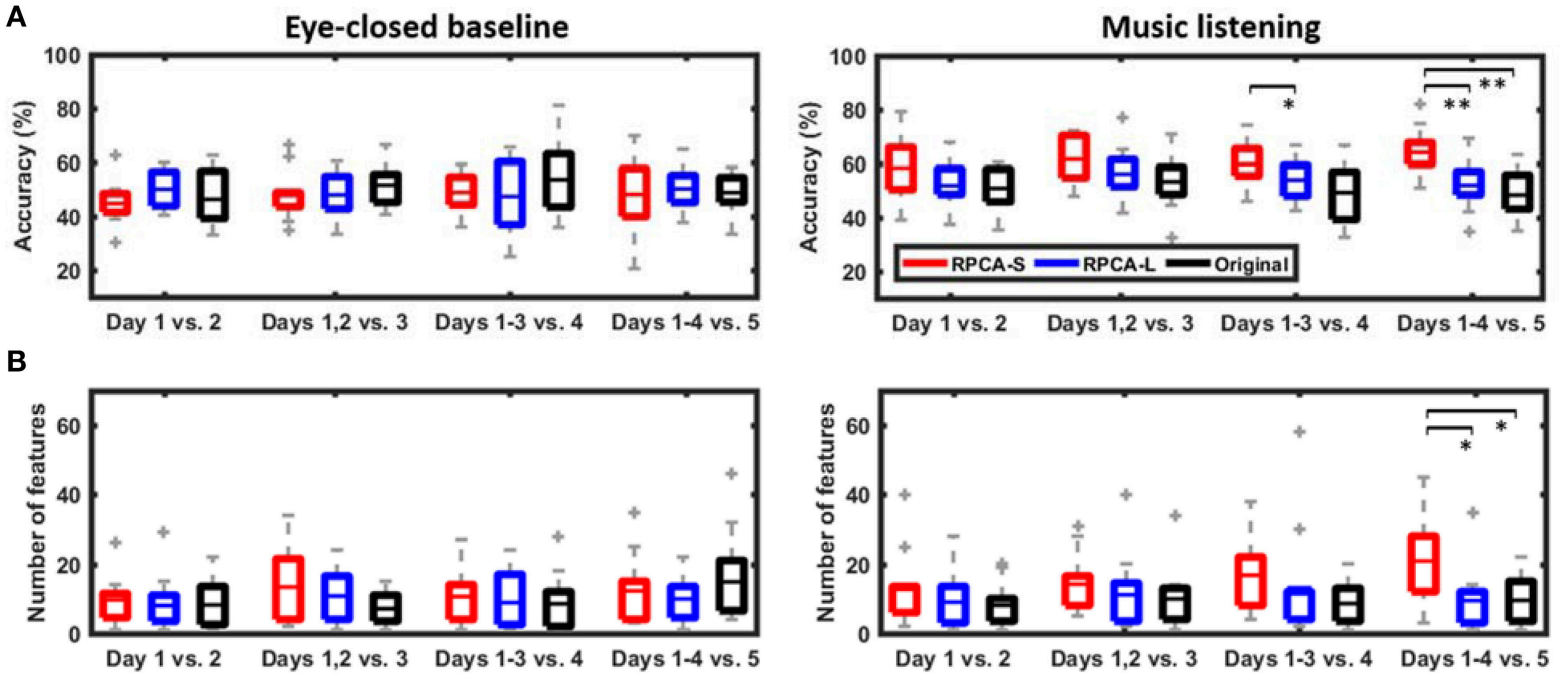
The cross-day emotion-classification performance in ADI manner. The performance included **(A)** the classification accuracy and **(B)** the explored number of informative features using with and without RPCA framework (RPCA-S, sparse matrix; RPCA-L, low-rank matrix; Original, original signals). The classification task during music listening was replicated in eye-closed resting period regarded as benchmark. Note that for the classification purpose, a resting period artificially used the same emotion label as its subsequent music-listening trial. ^*^ and ^**^ Refer to a statistical significance *p* < 0.05 and *p* < 0.01, respectively, using a two-sided Wilcoxon signed rank test.

Figure 4 shows the cross-day emotion-classification performance with and without using the RPCA framework in the ADI manner. The classification performance includes the number of informative features and the binary classification accuracy explored during music listening vs. eye-closed resting. There were two main findings in the comparative results. First, the RPCA framework improved the cross-day classification accuracy. For the music-listening classification accuracy (right panel in Figure 4A), the RPCA-decomposed sparse matrix (RPCA-S, red box) improved the classification accuracy monotonically as more data were added from additional recording days (Day 1 vs. Day 2: 58.31 ± 12.33%, Days 1–2 vs. Day 3: 61.53 ± 8.62%, Days 1–3 vs. Day 4: 59.65 ± 8.00%, and Days 1–4 vs. Day 5: 64.03 ± 8.40%). Such improvement was up to around 6% (*p* = 0.09 using a two-sided Wilcoxon signed rank test) in the case of four training days. In contrast, the RPCA-decomposed low-rank matrix (RPCA-L, blue box) and the original EEG signals without RPCA preprocessing (Original, black box) did not replicate such improvement along ADI conditions. Their accuracies were apparently worse than those of RPAC-S. There was a statistically significant difference between RPCA-L and RPCA-S for Days 1–3 vs. Day 4 (*p* < 0.05) and between RPCA-L/Original and RPCA-S for Days 1–4 vs. Day 5 (*p* < 0.01), respectively. Opposed to the above music-listening outcomes, the benchmark of eye-closed resting (left panel in Figure 4A) neither exhibited distinct differences with and without RPCA preprocessing within each ADI condition nor led to a tendency in classification accuracy along ADI conditions.

Second, the RPCA-S framework advanced the exploitation of informative features related to emotional responses. For the music-listening scenario (c.f. right panel of Figure 4B), the number of feature explored in RPCA-S was found to augment steadily as pooling EEG signals from one to four recording days (Day 1 vs. Day 2: 12.67 ± 5.99, Days 1–2 vs. Day 3: 14.17 ± 8.43, Days 1–3 vs. Day 4: 16.75 ± 5.74, and Days 1–4 vs. Day 5: 20.83 ± 7.18). The maximal increment was up to around 8 features for the ADI condition of four training days (*p* = 0.06). Unlike RPCA-S, both RPCA-L and Original exhibited features independent of the ADI conditions and typically yielded fewer features. Both of them were found significantly worse than RPCA-S for Days 1–4 vs. 5 (*p* < 0.05). For the eye-closed resting scenario, the number of features in RPCA-S tended to be comparable to those of RPCA-L and Original in each ADI condition and be independent to ADI conditions.

In sum, the RPCA-S led to progressive improvements in classification performance in terms of the number of informative features and the cross-day classification accuracy as long as the EEG signals leveraged from more recording days. This only worked for the music-listening scenario.

## Discussion

The present work studied how the inter-day EEG variability of emotional responses can be mitigated to facilitate cross-day emotion classification task, which was largely overlooked in the literature. This study extended our early proof-of-concept work (Jao et al., 2015) to validate the capability of the proposed RPCA-based signal filtering framework from the neurophysiological and realistic BCI perspectives through a five-day EEG dataset of 12 subjects. We first validated that the RPCA-decomposed sparse signals returned representative EEG features reflecting emotional responses that were relatively consistent across days. We further demonstrated that such sparse signals helped a machine-learning framework to exploit more informative features and lead to a progressive improvement on cross-day emotion-classification accuracy as EEG signals were engaged from multiple-day sessions. In contrast, neither its accompaniment low-rank signals nor the raw EEG features (i.e., without RPCA preprocessing) could replicate the above cross-day classification outcomes. The following sections discussed the RPCA findings upon the neurophysiological validity and machine-learning practicability.

### Neurophysiological validity underlying RPCA in emotion data

The present work aimed to exploit the underlying neurophysiological meanings associated with the two decomposed low-rank and sparse matrices using RPCA. By the mathematical definition of RPCA, the low-rank and sparse matrices account for the regular and sparse activities of the given streaming signals, respectively. The question herein was about what information the low-rank and sparse matrices actually account for in the EEG signals collected in an emotion-elicitation paradigm. There were three facets empirically indicating that the EEG oscillations captured in the sparse matrix were profitably linked to the implicit emotional responses. First, emotional responses elicited by music listening were considered as sparse activity. The induced EEG oscillations thus behaved as deviant activity to the concurrent intrinsic background activity, which presumably conformed to the mathematical role of the sparse matrix. As referred to the RPCA applications in other domains, the sparse matrix was also found to isolate sparse signal dynamics, such as, foreground moving objects in a video stream (Bouwmans and Zahzah, 2014), incoherent occlusion and disguise in a face image (Chen et al., 2012), neurocognitive lapses in driving (Wei et al., 2016), and a singing voice from music (Yang, 2012).

Second, the resultant music-baseline comparative outcomes of informative topographic feature maps (c.f., Figure 2) led to direct evidence. With respect to the sparse matrix, the low-rank matrix reciprocally dealt with regular activity in the given signals. Based on the-results of this study, the low-rank matrix was found to reveal less informative features as compared to the sparse matrix and its original input (without RPCA processing), yet tended to be marginally similar to the control benchmark of the eye-closed baseline scenario (Figures 2A,C). This thus implied that the low-rank matrix relatively summarized intrinsic background EEG activity with a minimal relationship with emotional responses, like the eye-closed resting. However, one may argue a few features remained in the analytical scenarios of the eye-closed baseline and the RPCA-L from the illustrated individual. This may be attributed to the fact that most of the eye-closed baseline periods were interleaved with music excerpts (c.f., Figure 1). The lingering emotion effects (Eryilmaz et al., 2011) might occur in our study. That is, the transit emotional responses induced in a regular music excerpt may remain and modulate the brain activity in subsequent resting state. In addition, as RPCA essentially involves a convex optimization problem (Candes et al., 2011), it may not lead to a perfect matrix-factorization decomposition. Some sparse activity may thus leak into the low-rank matrix (Han et al., 2017), contributing some minor information. Most critically, unlike the low-rank matrix, enormous emotion-relevant features emerged in the sparse matrix, which behaved most distinctly to the eye-closed condition.

Third, the music-baseline comparative cross-day classification performance in accuracy and the number of informative features (c.f., Figure 4) presented another conclusive evidence. The eye-closed resting periods barely provided discriminative information to conduct a binary emotion-classification task (i.e., around chance level) regardless of which analytical strategy (especially for the sparse matrix) was used and how much EEG-recording days were leveraged. Instead, the sparse matrix only worked valid for the EEG signals recorded in the music-listening period. Given more training days, the progressively increased number of informative features and the cross-day emotion-classification accuracy evidently inferred the discriminative yet emotional information exclusively captured by the sparse matrix.

### Impact of the inter-day EEG variability

Through the assessment to a five-day EEG dataset, the original EEG distributions (without RPCA processing) between training and test data across days (c.f., Figure 3) were found to be quite different. The binary clusters from an unseen day happened to be misleading or even reversal with the pre-learned class clusters, which exactly replicated the outcomes in Picard et al. (2001) and Christensen et al. (2012). Because of such the inconsistent class distributions, a classifier trained on one day may perform poorly on the test data collected from the same subject on another day. The resultant cross-day classification performance (c.f., Figure 4) reflected the negative impact of the inherent inter-day EEG variability, where involving more cross-day training sessions helped neither for exploring more robust informative features nor for optimizing the discriminative decision boundaries to yield a better classification accuracy. This implied that performing a multiple-day EEG collection and analysis barely worked without an efficient way to deal with day-to-day variability. It is worth noting that the aforementioned phenomenon may not emerge if an offline validation was adopted. For example, some works (Christensen et al., 2012; Liu et al., 2016) computed the cross-day classification accuracy by averaging the classification outcomes of all possible combinations of training and test days. Without a constraint on time ordering (e.g., allowing using Days 4, 5 to predict Day 1), the chance for including a day(s) having feature distributions compatible to those of a test day(s) likely increases (Lin et al., 2015). The plausible discrepancy of feature distributions on separate days and the corresponding degradation in cross-day classification accuracy might thus be overlooked. Furthermore, such an offline manner is unlikely the case for an ecological, real-life scenario, i.e., we may not use the data of a later day(s) to predict the emotional responses on a prior day(s). In contrast, the ADI scheme adopted by the present work considers time ordering and facilitates more realistic assessment of the impact of inter-day EEG variability.

The RPCA-based signal-filtering framework was proposed in the present work for the above purpose. Following the demonstrated neurophysiological evidence underlying the RPCA (see detailed discussions in the last subsection), the RPCA-decomposed low-rank matrix predominantly accounted for the background EEG activity that seemed to contribute more to the concerned inter-day EEG variability. That is, the RPCA-L and Original (before RPCA processing) compared favorably in cluster distributions along the ADI manner (c.f., Figure 3). Our exploratory results were in line with the previous outcome in motor imagery study (Shenoy et al., 2006), in which different background EEG activity was reported to shift the data in the feature space. After mitigating the background EEG perturbations (c.f., Figure 4), the phenomenon gave a direct support to the outcome of the improved cross-day classification accuracy in the RPCA-decomposed sparse matrix (i.e., RPCA-S). The extent of the improvement in accuracy when given more training days was attributed to the fact that the RPCA-S elaborated the class clusters gently yet progressively. Accordingly, the class distributions and the cross-day classification performance empirically demonstrated the posed hypothesis that the more the intrinsic EEG perturbations can be alleviated in each day, the more the elicited emotion-related EEG oscillations can be revealed.

This work has a limitation in elucidating plausible causes contributing the background EEG perturbations in the analyzed dataset. Some studies (Shenoy et al., 2006; Ahn et al., 2016) mentioned that the mental states of the subjects, such as, mental fatigue, attention level, engagement to the task, and sleep quality, may alter EEG patterns. This thereby suggested that future emotion study may include a comprehensive behavioral and mental questionnaire along with emotional labels, facilitating a systematic assessment of realistic EEG oscillations of emotional responses.

### Comparison to previous works

This work performed a binary emotion-classification task dedicated to an ecologically cross-day EEG dataset (five days). The realistic ADI validation manner was adopted to obtain the cross-day emotion-classification performance, which can straightforwardly infer the practicality of the proposed RPCA framework toward real-life applications. The study results showed that the optimal binary classification accuracy (using the sparse matrix, RPCA-S, c.f., Figure 4) was improved steadily from 58.31 ± 12.33% to 64.03 ± 8.40% as leveraging more EEG signals from one to four recording days for training. Nevertheless, most of previously related works (Koelstra et al., 2012; Koelstra and Patras, 2013; Lin et al., 2014; Gupta et al., 2016), in which a binary task was also conducted on limited data trials (using a long-duration elicitation materials), performed the analysis on a single-day dataset only, so that the impact of the inter-day EEG variability was not considered. As such, this study cannot make a direct comparison to the previous works, but instead we summarized their reported within-day binary classification accuracies for reference as follows: 55.4~62.0% for different emotion categories (referred to their Table 7 in Koelstra et al., 2012), 63.5~71.5% for different categories and features (Koelstra and Patras, 2013 referred to their EEG results in Table 3), 67~76% for different categories and features (referred to their Figure 2 in Lin et al., 2014), and 58~69% for different categories and features (referred to their in Table 5 Gupta et al., 2016). It was expected that the within-day accuracies that were not negatively affected by the inter-day variability outperformed the cross-day outcomes. The resultant cross-day accuracies of 58.31~64.03% using different training days in the present study seemed justifiable.

## Conclusion

This study proposed a robust principal component analysis (RPCA)-based signal-filtering strategy and incorporated it into a machine-learning framework to improve cross-day EEG-based emotion-classification performance. Through applying a realistic add-day-in validation manner to a five-day EEG dataset of 12 subjects, this study first validated that the RPCA-decomposed sparse signals predominately captured the EEG oscillations of emotional responses that were relatively consistent across days, and suppressed the day-fluctuated background EEG perturbations in its accompaniment low-rank signals. By leveraging EEG signals from all four recording days for training and tested for the last unseen day, the maximal improvement in the number of informative features and the cross-day classification accuracy appeared up to ~8 and ~6%, respectively. The original EEG features (prior to RPCA processing) neither achieved the cross-day classification task (i.e., the accuracy was around chance level) nor replicated the encouraging improvement due to the inter-day EEG variability.

## Ethics statement

The study was conducted in accordance with the Declaration of Helsinki and approved by the local Ethics Committee of University of California, San Diego.

## Author contributions

YL conceived analytical hypothesis, performed data analysis and interpretation, and drafted/revised the work; PJ performed data analysis and interpretation, and revised the work; YY contributed to data interpretation and revised the work. All authors approved the work for publication.

### Conflict of interest statement

The authors declare that the research was conducted in the absence of any commercial or financial relationships that could be construed as a potential conflict of interest. The reviewer MS and handling Editor declared their shared affiliation, and the handling Editor states that the process nevertheless met the standards of a fair and objective review.
